# Development and Usability Evaluation of a Facebook-Based Intervention Program for Childhood Cancer Patients: Mixed Methods Study

**DOI:** 10.2196/18779

**Published:** 2020-07-28

**Authors:** Bu Kyung Park, Ji Yoon Kim, Valerie E Rogers

**Affiliations:** 1 College of Nursing Research Institute of Nursing Science Kyungpook National University Daegu Republic of Korea; 2 Department of Pediatrics School of Medicine Kyungpook National University Daegu Republic of Korea; 3 School of Nursing (retired) University of Maryland Baltimore Baltimore, MD United States

**Keywords:** pediatric cancer patients, childhood cancer, social network site, Facebook, usability

## Abstract

**Background:**

Childhood cancers previously considered to be incurable now have 5-year survival rates up to 84%. Nevertheless, these patients remain at risk of morbidity and mortality from therapy-related complications. Thus, patient education and self-management strategies for promoting a healthy lifestyle are of tantamount importance for improving short- and long-term health outcomes. A Facebook-based “Healthy Teens for Soaam” (a Korean term for childhood cancers) program was developed to help improve knowledge and self-management practices of teens with cancer related to their disease and treatment.

**Objective:**

The two-fold purpose of this usability study was (1) to describe the process of developing an 8-week Facebook-based intervention program for teens with cancer, and (2) to evaluate its usability to refine the program.

**Methods:**

Multiple phases and methods were employed to develop and evaluate the usability of the program. Study phases included: (1) needs assessment through focus group interviews and qualitative content analysis, (2) development of module content, (3) expert review and feedback on module content, (4) Facebook-based program development, (5) usability evaluation by heuristic evaluation, (6) usability evaluation by targeted end-user testing, and (7) modification and final version of the program. Usability of the final version was confirmed through feedback loops of these phases.

**Results:**

Based on 6 focus group discussion sessions, it was determined that teens with cancer were interested in seeing stories of successful childhood cancer cases and self-management after discharge, and preferred multimedia content over text. Therefore, each Facebook module was redesigned to include multimedia materials such as relevant video clips tailored for teens. Usability assessed by heuristic evaluation and user testing revealed several critical usability issues, which were then revised. Potential end users tested the final program and perceived it to be usable and useful for teens with cancer.

**Conclusions:**

To our knowledge, “Healthy Teens for Soaam” is the first Facebook-based intervention program for teens with cancer. We actively worked with current childhood cancer patients and survivors to develop and improve this program, achieved good usability, and met the expressed needs and preferences of target end users. This 8-week Facebook-based educational program for teens with cancer, developed as the first step of an upcoming intervention study, will be useful for improving knowledge and self-management strategies of teens.

## Introduction

Survival rates associated with childhood cancers continue to improve, partly due to advances in diagnostic techniques and treatment modalities along with clinical research [1]. According to the Surveillance, Epidemiology, and End Results Cancer Statistics Review, the 5-year survival rate of children with leukemia 0-19 years of age in the United States is 84.1% and the 5-year survival rate of patients with childhood brain and central nervous system cancers is 74.8% [2]. In South Korea, the 5-year survival rate among patients with childhood cancers overall was 81.7% in 2014 [3].

Despite improvements in survival, childhood cancer survivors still face a high risk of therapy-related complications or adverse effects that persist or arise after completion of treatment [4]. Childhood cancer survivors are at significantly increased risk of relapse, second malignancy, and long-term late effects, including cardiovascular and pulmonary dysfunction, endocrine disorders such as metabolic syndrome, and others [5,6]. Specifically, childhood cancer survivors of 5 years or greater have at least one chronic health condition [7]. A study on the late effects of childhood cancer treatment found that the cumulative incidence of a chronic health condition among long-term childhood cancer survivors was 99.9% by 50 years of age, with a nearly 2-fold greater cumulative burden among survivors compared to matched community controls [5].

Some morbidities are modifiable through preventive healthy behaviors such as physical activity, good nutrition, obesity prevention, and avoidance or cessation of smoking, as well as identification of characteristics that may modify morbidities or adherence to healthy behaviors to better target interventions [8,9]. For example, one study showed that childhood cancer survivors of older age and lower socioeconomic status were less frequently engaged in preventive healthy behaviors [10]. Another study reported that a greater proportion of female survivors smoked compared to teens without cancer [11]. Although childhood cancer survivors have been found to engage in unhealthy behaviors (eg, tobacco, alcohol, drug use, and sexual behaviors) and to be nonadherent to national health behavior guidelines at rates similar to those of their healthy siblings and teens without cancer [12,13], these behaviors are likely to be more consequential for individuals with organ damage secondary to cancer treatment. Moreover, late effects of treatment such as obesity, cancer-related pain, and sensory impairments have been significantly associated with increased risk of comorbid symptoms [14]. Increased comorbidities are associated with decreased quality of life, and with an increased risk of hospitalization and mortality [15]. Thus, the importance of patient education and promoting self-care for a healthy lifestyle for teens with cancer is increasingly becoming recognized to help mitigate complications of cancer and its treatment.

Children currently undergoing cancer treatment, as well as survivors, require follow-up care for the rest of their lives; thus, the Children’s Oncology Group (COG) has emphasized the importance of regular medical follow-up and has developed the “COG long-term follow-up guidelines for survivors of childhood, adolescent, and young adult cancers” [16]. In addition, various interventions to improve long-term outcomes for teens with cancer have been developed worldwide. For example, the Nursing Discipline of the COG has developed key principles and recommendations for patient and family education practices [17] as well as interventions for teens with cancer, such as nutrition and cooking workshops [18], nutrition and body weight changes [19], systematic intervention for psychological preparation for radiotherapy treatment [20], mobile health intervention to improve adherence and quality of life [21], and a web-based physical activity intervention [22]. However, few intervention studies involving teens with cancer have been conducted in Korea. Existing examples include web-based patient safety education [23], an art intervention for siblings of children with cancers [24], and educational interventions to enhance adherence to prophylactic treatment [25]. Intervention programs for preventing long-term late effects of childhood cancers and their treatment are glaringly absent. In particular, there is a crucial need for interventions that promote self-management strategies for achieving a healthy lifestyle among teens with cancer. This is important, as they will likely survive into adulthood and need to develop the knowledge and skills necessary to prevent or mitigate the late effects of cancers, and thereby improve their health-related quality of life.

Knowledge about their disease, the importance of treatment adherence, and strategies to improve adherence could be improved through innovative health interventions designed specifically for teens [21]. Among teens with cancer, inadequate information was identified as one of the barriers to compliance with chemotherapy [26]. Adult survivors of childhood cancers were found to lack detailed knowledge of their treatment history and risk for late effects [27]. Therefore, further efforts are needed to educate and empower teens with cancer to gain appropriate knowledge and assume responsibility for their health management [28].

Social media has been increasingly utilized as a platform for delivering health interventions in childhood cancer care. For example, Watson [29] reported that oncology health care professionals utilized social media to listen, learn, engage, and cocreate to advance cancer care. Teens with cancer have also turned to social media for information about cancer or to interact with peers and others about their diagnosis and its impact [30]. Importantly, the effectiveness of using social media platforms such as Facebook has been reported for this tech-savvy generation. For example, a previous study [31] found that a randomized trial of a Facebook-based physical activity intervention among young adult cancer survivors significantly increased their physical activity. Therefore, we developed an educational program for teens with cancer on Facebook called “Healthy Teens for *Soaam*” (*soaam* is the word for pediatric cancers in Korean), as the first step of an intervention that will deliver educational materials with the aim of increasing knowledge and improving self-care practices related to their disease and treatment. The purpose of this usability study was (1) to describe the process of developing this 8-week modular intervention program for teens with cancer and (2) to evaluate its usability to refine the program.

## Methods

### Study Design

**Figure 1:**
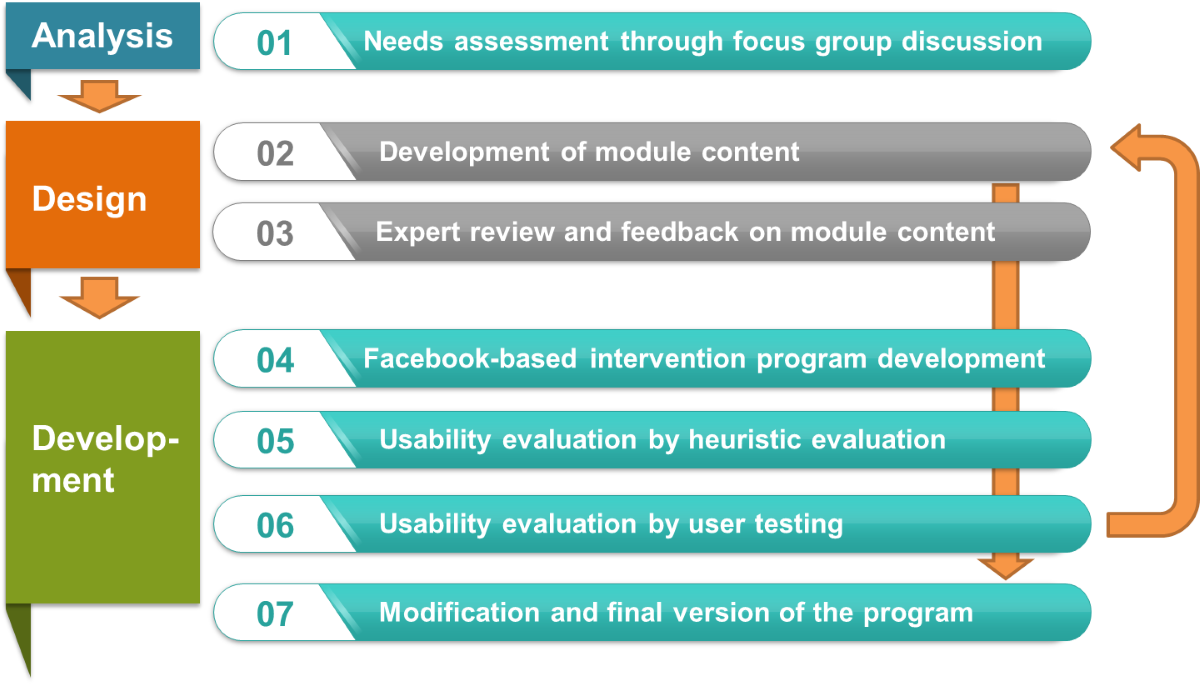
Flow chart of "Healthy Teens for Soaam" program development.

This study was reviewed and approved by the principal investigator’s institutional review boards (1041078-201606-HRSB-122-01 and 20200026). The study was conducted in 7 phases (Figure 1) and was guided by the Analysis, Design, Development, Implementation, and Evaluation (ADDIE) instructional design approach [32]. This article describes the first 3 phases of the ADDIE approach.

### Phase 1: Needs Assessment Through Focus Group Discussion and Qualitative Content Analysis

Semistructured focus group discussions were conducted with 12 teens with cancer. Participants were recruited from childhood cancer self-support groups in Korea using convenience and snowball sampling methods. A flyer was posted to the online platforms of self-support groups on popular Korean online platforms (Naver Café, KakaoTalk, and Band), and interested volunteers contacted the researchers by phone or email. Interested participants also suggested other potential participants to the researcher.

A total of 6 in-person focus group discussion sessions were conducted, during which participants were accompanied by their parent or legal guardian. Each session included 2 to 5 participants, and each lasted for about 60 to 90 minutes. Focus group discussions were facilitated by the principal investigator (BP) and a research assistant. First, the principal investigator explained the overview of the program, distributed handout materials detailing program contents, and asked participants and parents to provide their opinions regarding the contents. For example, they were explicitly prompted to provide suggestions regarding the addition, removal, or emphasis of content. Besides answering questions, participants and parents were encouraged to share their personal experiences with childhood cancer using prompts such as “If you have anything you want to share with us even though it is not directly related to the contents, don’t hesitate to tell us.” Focus group discussions were recorded and transcribed verbatim for qualitative content analysis using NVivo 11 (QRS International, Burlington, MA, USA).

#### Focus Group Discussion Data Analysis

Qualitative content analysis was conducted using a combination of the content analysis method suggested by Krippendorff [33] and the inductive coding approach suggested by Elo and Kyngäs [34]. Coders read the transcripts and freely generated themes. They then developed categories from similar themes as the coding progressed. Major coding rules included the following: (1) a category was created if a list of at least three themes was generated under that category, and (2) multiple sentences from the same participant that referred to the same content were coded as one unit.

Qualitative content analysis was conducted by the two coders (the principal investigator [BP] with a PhD in nursing and the research assistant with a master’s degree in nursing) to ensure reliability of the analysis. Coders independently analyzed the transcripts. Questions or disagreements regarding the themes or categories were discussed. Revision and refinement of themes and categories continued until content analysis was completed.

Based on results from focus group discussions, the program module content was organized using the following usability design methods. First, we used group information together (also known as card sorting) after the focus group discussions. We defined the important themes from the focus group discussions and grouped them into several categories such as diagnosis, examination, treatment, social support, and return to society/school. Second, we developed a structure for 8 weekly modules. Third, we developed low-fidelity module prototypes such as paper prototypes. Finally, we established high-fidelity mockups using PowerPoint slides.

### Phase 2: Development of Module Content

Main topics for the program module content were developed based on the results from the focus group discussion “needs assessment.” Module content was written at a sixth-grade reading level using the Flesch–Kincaid readability test guidelines [35,36]. Content was developed with guidance from published research articles, medical and nursing textbooks, information available from health professional organization websites (eg, Korean Association for Children with Leukemia [37]), patient education materials in hospitals, and consultations with a panel of experts.

We incorporated usability principles for teen-friendly websites [38] into the program prototype in terms of content, appearance, and navigation. For example, we developed the content using PowerPoint 2016 (Microsoft Corp, Redmond, WA, USA) slides visualized on a single page without scrolling [38,39]. To improve appearance, we used large font sizes and avoided garish color schemes [38,39]. Moreover, slides for each module had their own theme to distinguish them from other modules. Although Facebook posts appear in reverse chronological order (ie, the most recent post appears on the top of users’ News Feeds), this was not considered a potential problem for participants because the module content was designed to be updated weekly and potential target participants were familiar with Facebook navigation.

Each module included specific learning objectives, structured learning material for fundamentally important content with external web links for further information, multimedia such as video clips or games relevant to the topics, and discussion topics at the end of the modules. Participants were asked to respond to discussion topics by adding comments on Facebook. This served to monitor module participation and sharing of personal experiences for social support. Although not done during testing, in the implementation study, a program moderator (researcher) will provide feedback and encourage interactions among participants on discussion boards. Discussions included the following topics: “Let’s talk about blood transfusion experiences. Have you ever received a blood transfusion? If yes, please tell us about the specific blood product you received, your feelings, and side effects,” and “Have you had any pain while getting treatment in the hospital? Which one was the most painful, and how did you overcome the pain—pain medication, massage, and ice bag?”

### Phase 3: Expert Review and Feedback on Module Content

The module content was reviewed by four experts currently working in pediatric hematology and oncology units in tertiary hospitals, who each had more than 10 years of experience: two nurses with master’s degrees in pediatric nursing and two pediatric hematology and oncology physicians with PhDs. Reviewers were knowledgeable about the most recent evidence in this highly specialized field and reviewed the module contents for accuracy. They were asked to rate the ease of understanding and appropriateness of information for teens with cancer (1=strongly disagree; 2=disagree; 3=agree; 4=strongly agree). Based on their feedback, module contents were revised and finalized for mockups of the Facebook-based program.

### Phase 4: Facebook-Based Intervention Program Development

Facebook Group was used to develop the 8-week intervention program, with the “private group” functionality utilized to protect participants’ privacy. With a private group, only people who are invited by the group creator (principal investigator of this study) can join and see who is in the group and what they post [40]. We chose the private group option because, as established by the focus group discussions, many participants did not want to be widely known as cancer patients. Additionally, we customized the group’s privacy options [41], for example, by selecting the “hide group” and “membership approval” options so that only members could find the group and that only the group creator could approve new members.

To deliver module content, we composed PowerPoint slides and then uploaded each slide to the group’s Photos section. Participants could easily navigate the slides by clicking the “next” and “back” buttons. Considering that the purpose of this program is to deliver accurate and reliable educational content, only group administrators were granted posting permission, but participants were allowed to add comments to each post to share their opinions or contribute to discussions.

### Phase 5: Usability Evaluation by Heuristic Evaluation

Two experts in the field of usability and human-computer interactions reviewed the Facebook-based program using Nielsen’s heuristics [42,43] adapted and tailored for children’s electronic learning (eLearning) program evaluation [44]. The two usability experts have PhDs in health care informatics and were currently teaching and conducting research on health care informatics in universities, one for 17 years and the other for 5 years. The criteria of Nielsen’s heuristics tailored for eLearning in children were: (1) visibility of system status; (2) match between system and the real world; (3) user control and freedom; (4) consistent and standards; (5) error prevention; (6) recognition rather than recall; (7) flexibility and efficiency of use; (8) esthetic and minimalist design; (9) help users recognize, diagnose, and recover from errors; (10) help and documentation; (11) design attractive screen layout; (12) use appropriate hardware devices; (13) challenge the child; (14) evoke child mental imagery; and (15) support child curiosity [44]. Comments from the heuristic evaluation were analyzed employing content analysis using the inductive coding approach described in Phase 1. The program was revised according to feedback from the heuristic evaluation.

### Phase 6: Usability Evaluation by User Testing

User testing was conducted using observation, the think-aloud method, voice and screen activity capture using Camtasia 9 (TechSmith, Okemos, MI, USA), and surveys. Participants for this phase were recruited through parents of teens belonging to a childhood cancer self-support group in Korea using convenience and snowball sampling. A flyer was posted to the online platforms of self-support groups on KakaoTalk and Band, and interested volunteers contacted researchers by phone or email. Inclusion criteria were teens with cancer who were (1) aged 13 to 18 years old, (2) diagnosed with any type of childhood cancer, (3) received any type of cancer treatment (eg, radiation therapy, chemotherapy, stem cell transplant), and (4) at any stage of treatment (newly diagnosed through completed treatment). Participants arranged appointments for screening, consenting, and user testing. As the participants were adolescents, both informed consent from their parents or guardians and assent from the teens were obtained prior to participation.

#### Procedures

A total of 11 face-to-face, 1:1 user testing sessions were conducted. Testing was facilitated by the principal investigator in a private room at the study site. Before conducting user testing, the researcher gave instructions about the program and usability evaluation methods such as the think-aloud method and Camtasia screen-capture software. Participants were then provided with an information page that included the Facebook program link, user ID, and password. They were assigned a user testing task to review 2 out of the 8 modules, including logging in, navigating through instructions on the first page to modules, reading content, watching video clips, and adding comments. They were assured that they could stop at any time for any reason. Each session lasted 30 to 40 minutes.

During evaluation of the program, participants were encouraged to think out loud while their voices were recorded using Camtasia. During pauses from thought verbalization, participants were probed with the following prompts: “What do you think about this? What features do you think need improvement?” At the same time, the researcher documented comments from participants and her own observations using the user testing worksheet.

#### Measures

Before user testing, participants’ demographic characteristics as well as their internet and Facebook usage habits were queried and documented. After user testing, participants filled out a short survey that evaluated electronic health (eHealth) literacy and perceived usability. The eHealth literacy was assessed using the eHealth Literacy Scale (eHEALS) [45], which has 8 items and assesses an individual’s knowledge, comfort, and perceived skills for locating, evaluating, and applying eHealth information for health issues. The Korean version of eHEALS, known as K-eHEALS, was previously tested for reliability and validity [46]. Total eHEALS scores range from 8 to 40, with a higher score indicating better health information literacy. Cronbach α of the original scale was .88 [45] and was also .88 for the Korean version [46]. Cronbach α for this study was .71.

Perceived usability of the program was assessed using the Perceived Health Website Usability Questionnaire (PHWSUQ) [47]. This questionnaire has 12 items scored using a 7-point Likert scale. It measures three dimensions of usability: satisfaction, ease of use, and usefulness. Cronbach α values for the subscales ranged from .64 to .93, and was .85 for this study.

#### Data Analysis

Quantitative data from the questionnaires were analyzed with descriptive statistics using SPSS Statistics for Windows, version 25.0 (IBM Corp, Armonk, NY, USA). Audio recordings from user testing sessions were transcribed verbatim and analyzed employing the content analysis method described in Phase 1. Qualitative data, collected throughout observation and screen activity and recorded by Camtasia, provided a rich contextual background and a strong source of triangulation for developing themes and generating a comprehensive review of the program’s usability. Thus, screen recordings were reviewed when the transcript did not capture enough usable detail, which improved the accuracy of content analysis.

### Phase 7: Modification and Final Version of the Program

Modifications regarding content and appearance issues were made on the PowerPoint slides, and the revised slides were uploaded to Facebook (the process was conducted in the order phase 2 to phase 4 to phase 7). If content revisions required expert opinions, relevant experts were contacted to confirm the accuracy of the new content (the process was conducted in the order phase 2 to phase 3 to phase 4 to phase 7).

## Results

### Phase 1: Summary of Focus Group Discussions

Participant characteristics are summarized in Table 1 [48].

Participant feedback regarding the outline of the program (Table 2) included (1) a desire for stories of successful cases and self-management at home, (2) a preference for multimedia content (eg, video clips) rather than text, (3) requests for more patient safety–related material, (4) an expressed need for detailed program objectives and instructions, and (5) appeals for more information on treatment, prognosis, and medical terminology. One teen expressed their support for the program as follows:

This is the big problem. The reason I’m so supportive of this education program (Healthy Teens for Soaam) is that there’s a limitation of internet and you narrow down the range a little bit…... I hope the contents go on in the future.

Additionally, participants freely described and shared their experiences with cancer treatments, and the emerging themes and categories dealt with information needs, support systems, barriers to treatment, facilitators to treatment, return to social life, and health care system issues. Other representative comments from the focus group discussions are provided in Multimedia Appendix 1.

**Table 1:** Characteristics of focus group discussion participants (N=12).

Characteristic		Value	
Age (years), mean (SD)			15.2 (5.3)
**Sex, n (%)**			
	Male		6 (50)
	Female		6 (50)
**Diagnosis, n (%)**			
	Leukemia		6 (50)
	Brain tumor		2 (17)
	Aplastic Anemia		2 (17)
	Other		2 (17)
**Treatment, n (%)**			
	Completed with ongoing outpatient follow up		9 (75)
	Ongoing chemotherapy		3 (25)

**Table 2:** Content analysis of focus group discussion transcriptions (N=12).

Theme		Frequency of units
**Feedback on the program**		
	Add story of successful cases and self-management after discharge	9
	Prefer multimedia (video clips) than text	10
	Add patient safety issues	11
	Need detailed program objectives and instructions	13
	Add information on treatment, prognosis, medical terms	20
**Information needs**		
	Lack of information or inaccurate information	7
	Current sources of information (eg, other patients, hospital handout, searching the internet)	19
	Useful information during treatment (personal experience)	20
**Support system**		
	Current support from hospital, school, pediatric cancer associations, self-support groups, etc	10
	Need systematic supporting system for patients and families	13
	Useful support during treatment (personal experience)	15
**Barriers to treatment**		
	Economic problems	5
	Hospital-related issues (eg, lack of available beds for admission, manpower shortage, miscommunications between health care providers)	8
	Side effects of treatment (eg, chemotherapy, transfusion, infection)	25
	Emotional reactions at the time of diagnosis and during treatment	28
**Facilitators to treatment**		
	Patients’ insight on diagnosis	4
	Empathy of health care providers	6
	Social support from friends, other patients, family members	13
**Return to social life**		
	School life	4
	Lack of physical activity	6
	Concerns related to infection prevention, weak immunity	10
**Health care system issues**		
	Disabled child registration and welfare benefits	2
	Inadequate and poor social welfare system	4
	Health insurance	5

### Phases 2 and 3: Facebook-Based 8-Week Intervention Program

An overview of the final version of the program is provided in Table 3. Content was revised and updated according to focus group discussion feedback. Participants and parents were interested, for example, in the topics of returning to school and the effects of chemotherapy on the adolescent’s fertility; therefore, we added these topics to the modules. Additionally, as participants preferred multimedia over reading text, we searched for movies and Korean dramas depicting stories involving teens with cancer and included links to short video clips in the relevant module content. Animations explaining cancers and their treatment tailored for teenagers were also added for a better understanding of how treatment proceeds.

Four childhood cancer experts reviewed the content and provided feedback on whether the information was accurate and current, including the pictures and video clips from movies and Korean dramas. Two nurses reviewed relevant medical terminology from academic journals and textbooks, and noted that it differed from the terminology commonly used by participants and parents. For example, granulocyte colony-stimulating factor injections for neutropenia were usually referred to as “count shots” (ie, a shot given when the absolute neutrophil count is low) or “Grasin” among participants and parents. Thus, we also used these words in the program content. Two physicians contributed information on the most recent treatment practices for childhood cancers in tertiary hospitals. For example, a previous version contained only bone marrow transplantation, but we added information about cord blood transplantation and peripheral blood stem cell transplantation.

**Table 3:** Overview of the Facebook-based Healthy Teens for Soaam program.

Module (Week)	Contents
Introduction	Program purpose, program schedule, how-to-use tutorial video clip
Module 1 (Week 1)	Pediatric cancers characteristics -Pediatric cancers statistics (risks and causes of pediatric cancers) -Pediatric cancers symptoms, early signs -International Childhood Cancer Day (February 15) -Psychosocial services and support for children and families
Module 2 (Week 2)	Types of pediatric cancers and their characteristics -Bone cancers, brain cancers, leukemia, hepatoblastoma, lymphoma, neuroblastoma, rhabdomyosarcoma, retinoblastoma, Wilms tumor, etc
Module 3 (Week 3)	Diagnostic tests -Imaging tests, CT^a^ scans, MRI^b^, ultrasound, blood tests -Bone marrow aspiration and biopsy
Module 4 (Week 4)	Pediatric cancers treatments 1 -Hickman catheter insertion and management -Chemo-port insertion and management -Radiation treatment and chemotherapy
Module 5 (Week 5)	Pediatric cancers treatment 2 -Treatment team and frequently used medical terminology -Caring tips for mouth care and oral mucositis -Caring tips for infection and bleeding -Symptoms of infection, ANC^c^
Module 6 (Week 6)	Pediatric cancers treatment 3 -Managing side effects of radiation and chemotherapy -Pain, transfusion -Growth and development, fertility -Stem cell transplantation (BMT^d^, CBT^e^, PBSCT^f^)
Module 7 (Week 7)	Back to school and society -Follow-up care after cancers treatment -Facilitating school reentry guide -Nutrition and daily activity
Module 8 (Week 8)	People around me: family and friends Review and summary

^a^CT: computed tomography.

^b^MRI: magnetic resonance imaging.

^c^ANC: absolute neutrophil count.

^d^BMT: bone marrow transplantation.

^e^CBT: cord blood transplantation.

^f^PBSCT: peripheral blood stem cell transplantation.

### Phases 4 and 5: Revision After Heuristic Evaluation

Usability experts pointed out usability problems of the program among three categories of Nielsen’s heuristics tailored for children’s eLearning [44]: (3) user control and freedom, (7) flexibility and efficiency of use, and (10) help and documentation. For example, even though Facebook has a Help menu for user support, the experts suggested that there should be a Help section specific to the program. Therefore, we added a “Help and FAQs” section for dealing with problems encountered while using the program. However, some usability issues were not changeable on the ready-made Facebook platform; these included Facebook advertisements and the reverse chronological order of posts.

All four childhood cancer experts rated the content highly for its ease of understanding (4=strongly agree) and appropriateness for teens with cancer (4=strongly agree). They also commended the detailed instructions and the neat and consistent overall design of the PowerPoint slides.

### Phases 6 and 7: Revisions After User Testing

User testing participant characteristics (Table 4) included a mean age of 16.7 years, and the majority had leukemia and were Facebook users (91%). Their mean K‑eHEALS score suggested good eHealth literacy. The mean PHWSUQ score (reflecting the program’s perceived usability) indicated that the program was perceived as usable. All 11 participants completed the user testing tasks and 9 participants were able to navigate through the program with little or no guidance. The mean testing time was 38.8 (SD 3.1) minutes. Representative quotes from user testing are shown in Multimedia Appendix 1.

**Table 4:** Characteristics of user testing participants (N=11).

Characteristic		Value
Age (years), mean (SD)		16.7 (1.1)
**Sex**		
	Male	7 (64)
	Female	4 (36)
**Diagnosis**		
	Leukemia	8 (73)
	Other	3 (27)
**Facebook user**		
	Yes	10 (91)
	No	1 (9)
K-eHEALS^a^, mean (SD)		27.6 (1.3)
**PHWSUQ^b^, mean (SD)**		
	Total PHWSUQ	60.1 (2.8)
	Satisfaction	32.9 (4.8)
	Ease of use	15.4 (2.9)
	Usefulness	17.8 (3.4)
**Preferred frequency of content update, n (%)**		
	Every day	1 (9)
	2-3 times per week	4 (6)
	Once per week	6 (55)

^a^K-eHEALS: Korean version of the eHealth Literacy Scale (range 8-40).

^b^PHWSUQ: Perceived Health Website Usability Questionnaire (range 12-84 total; 6-42 satisfaction; 3-21 ease-of-use and usefulness).

The transcript of user testing content analysis identified 20 themes under four categories: program content, program appearance, navigation, and others, which mainly included feedback about the strengths of the program (Table 5). Participants mentioned that they gained some new knowledge from this program, but some perceived the content as difficult to understand or as having low readability because of the medical terminology. Regarding the appearance of the program, participants generally liked the layout, but three users noted, for example, that some text was hidden behind images or that low-resolution figures were difficult to decipher. Nine participants had no issues with navigation of Facebook, but two participants needed help from the researcher. Seven participants expressed their impressions about the discussion section, where they could share personal experiences with participants who had similar experiences. For example, one teen said: “Then if I don’t know the details right now, I think it would be helpful for patients to learn this by Facebook myself.”

Critical usability issues reported during user testing were reviewed, and, where necessary, revisions were made and confirmed through feedback loop of the phases (Figure 1). Regarding usability issues with “program content,” one participant suggested a movie on a pediatric patient, which was not in our movie list. She mentioned that it had helped her a lot when she underwent treatment. In this case, childhood cancer experts reviewed the movie and included relevant video clips to the module. Some video clips and pictures that were not working or had low graphic resolution were replaced with other video clips and pictures. Usability issues with “program appearance” were mostly minor revisions such as typos, font size, or picture location issues, which were corrected directly on the PowerPoint slides, and then the revised slides were reuploaded on Facebook. There were few usability issues with “navigation.” Participants easily recovered from failed paths, with brief help from the researcher. However, for first-time users of Facebook, we added a short video tutorial of the program navigation. Multimedia Appendix 2 shows a screenshot of the final version of the “Healthy Teens for *Soaam*” program on Facebook.

**Table 5:** Content analysis of user testing transcriptions (N=11).a

Category, Themes			Type of feedback	Frequency of units
**Program Content**				
	Reading level of elementary or middle school		Positive	2
	Explanations on medical terminologies are useful		Positive	7
	Video clips and pictures were helpful		Positive	14
	Easy to understand, good readability		Positive	28
	Helpful and useful educational content		Positive	34
	Knowledge/information first learned from this program		Positive	35
	Need revisions or irrelevant information		Negative	2
	Some video clips and pictures were not working or had low graphic resolution		Negative	3
	Need more information on specific topics		Negative	5
	Content was not easy to understand, low readability (use of difficult medical terminology)		Negative	7
**Program Appearance**				
	Liked layout (eg, font style, font size, sentence length, location of pictures and paragraphs)		Positive	23
	Suggestions		Negative	3
	Issues with layout (eg, picture sizes and locations)		Negative	6
**Navigation**				
	Familiar with Facebook navigation		Positive	6
	Got lost or failed path (needed help from the researcher)		Negative	2
**Others (strengths of program)**				
	Reasons for not doing or following self-management at home		Positive	3
	Provide online social support		Positive	4
	Good sources of information: from health care professionals, school, the internet, family, self-experience		Positive	14
	Improved intention (attention) to know about their disease		Positive	15
	Can share personal experiences on treatment		Positive	17

^a^Representative comments from user testing are shown in Multimedia Appendix 1 (quotations from focus group discussions and user testing).

## Discussion

### Principal Findings

The aim of this study was to develop a Facebook-based intervention program for childhood cancer participants and to evaluate its usability to guide program refinements. Overall, the evaluation of “Healthy Teens for *Soaam*” revealed that the program was perceived as usable by our participants, who were representative of our target audience (teens with cancer). Seven phases of development and usability evaluation uncovered usability issues as well as areas to enhance user satisfaction, which were then modified accordingly.

Childhood cancer participants and families wanted a comprehensive online information source where they could find childhood cancers–related information. Preexisting sources of information (eg, other patients, hospital handouts, searching the internet) were not reliable and potentially provided limited or outdated information. Therefore, focus group discussion participants greatly valued the objectives and content of this program. Moreover, participants supported the discussion topics at the end of each module. Considering the characteristics of adolescence, peer groups are important for their socialization; however, hospital admissions and principles to prevent infection limit their opportunities to participate in peer group activities. Online interactions among teenagers with similar diagnoses provide the next-best opportunity for social support.

User testing by potential end users revealed that the program content was comprehensive. Previous research has found the two main predictors of noncompliance to chemotherapy to be child resistance and inadequate information [26]. To overcome these barriers, this program provided tailored information to teens with the goals of improving their knowledge and reducing their resistance to treatment by explaining the rationale behind treatments and procedures, and may therefore improve treatment compliance among teens with cancers.

By adapting the Facebook platform to this program, we took advantage of the following key points: (1) potential end users are familiar with navigation of the program, (2) researchers can reduce costs for developing new online platforms, and (3) researchers may be able to improve treatment fidelity, as teenagers access Facebook almost every day and are continuously connected via the smartphone app. By contrast, the limitations of using Facebook [49] include: (1) design-related limitations (eg, limited freedom and options for text editing and background color schemes) and (2) the inability to control certain features such as advertisements and chronology of posts.

### Next Steps

The next step of this “Health Teens for *Soaam*” program will be delivering this 8-week Facebook-based intervention for teens with cancer in South Korea. The purpose will be to increase knowledge and provide accurate, up-to-date information about self-management strategies for improving health and wellness. Through this intervention, we will be able to determine the effectiveness and efficiency of the Facebook-based intervention program.

A previous Facebook-based intervention program for young adult cancer survivors found that engagement with the Facebook program was variable, and investigators recommended that future research should explore how to promote sustained engagement in online social networking [31,50]. Our program includes discussion sections similar to the previous intervention [31,50], in that relevant discussion topics are included in each module. To foster interaction and social support between participants, a moderator will encourage and remind users of discussion sections. A review of the use of social media for teens with childhood cancer [30] reported that health care providers are increasingly integrating social media into their professional life, and that it provides several advantages for both patients and health care providers. For example, the presence of health care providers presents an opportunity for direct interaction with patients and the ability to provide them with reliable, data-based health information. In turn, the health care providers also learn about the experiences and concerns of teens with cancer in real time. Thus, we will consider inviting pediatric oncology health care providers to serve as additional moderators who can provide expert opinions in response to participant queries. This option will improve our study, promote better participant engagement, and enhance treatment fidelity.

### Strengths and Limitations

This study had several strengths. We used rigorous qualitative analysis methods, including focus group discussions and user testing transcriptions. Additionally, the use of multiple methods provided a strong source of triangulation and enhanced the reliability of our results, which were informed by both qualitative and quantitative data. We described all development and usability evaluation processes conducted in 7 phases to provide guidance to researchers who want to use Facebook as an intervention platform. According to a recent systematic review of the use of Facebook, only 10 studies have investigated this platform for such interventions [51]. This could be partly because there is no guide on how to utilize this platform, and the task could seem daunting without guidance. Lastly, we used multiple usability evaluation methods. For example, we relied on expert reviews of our content by health care professionals, heuristics evaluation of the Facebook program by human-computer interaction experts, and user testing of the Facebook program by potential end users. Feedback comments from the different evaluation methods guided different aspects of usability issues and improved various facets of our program.

Our study also had several limitations. Regarding participant recruitment, focus group discussions and user testing employed convenience and snowball sampling methods. Sample sizes were relatively small and participants were from one metropolitan area. We did not apply strict inclusion criteria for user testing participants, instead including participants with any type of cancer and at any stage of treatment (ranging from newly diagnosed to completed chemotherapy). Additionally, the current health status of participants was biased toward teens who were in good health. These limitations could decrease the generalizability of the study findings. In addition, our findings may reflect a response bias, as participants who were already interested in Facebook may have been more likely to participate.

## Appendix

Multimedia Appendix 1 Quotes from focus group discussions and user testing sessions.

Multimedia Appendix 2 Screenshots from the Healthy Teens for Soaam program.

## Abbreviations

ADDIE: Analysis, design, development, implementation, and evaluation
COG: Children’s Oncology Group
eHEALS: eHealth Literacy Scale
eHealth: electronic health
eLearning: electronic learning
K-eHEALS: Korean version of the eHealth Literacy Scale
PHWSUQ: Perceived health website usability questionnaire
