# Supplementary material for: Development and Usability Evaluation of a Facebook-Based Intervention Program for Childhood Cancer Patients: Mixed Methods Study
Source: J Med Internet Res. 2020 Jul 28;22(7):e18779. doi: 10.2196/18779 (PMC7420636; doi:10.2196/18779)
Supplement: Multimedia Appendix 1 [file jmir_v22i7e18779_app1.pdf]

Appendix 1. Quotations from focus group discussions and user testing sessions

I. Focus group discussion: categories, themes, and feedback

1. Feedback on the program: Add information on treatment, prognosis, medical terms

1) Treatment

"What I care about is if I am going to get surgery, and if I need continuous chemotherapy treatment. Also, I am curious about all these treatment procedures, and what will happen after a leg amputation."

"In many cases of osteosarcoma, you take an x-ray in a local clinic and just continuously take physical therapy, and then finally move to a big hospital, usually because the physicians misread the x-ray. So, it will be better if you attach a picture that can compare to my x-ray. Patients can also recognize the difference between normal bones and bones which have osteosarcoma, and come to know their bones need to have further tests."

"Well, I was very shocked; I didn't know (how a bone marrow transplant progresses). People who don't know exactly what it is, they will be shocked, too."

"(A parent said) And that is the biggest point; in the case of aplastic anemia like ours, I think it would be good to show us briefly so that we can see how it's done, because no one gives an explanation.… it was like that at the time. It's easy for the kids not to be afraid of the transplant scene, but once they go in for a transplant, they get really nervous. They get air-showered and go in, then they find a nurse in there and feel anxious and terrified. If you tell them how the transplant goes and here, this place is a sterile room,

which you must keep some promises to protect some things with ease and fun, talking about other things, it would be good in the perspective of consumers. But, speaking from an expert's point of view, it's burdensome and frightening."

2) Prognosis

"How you would get treatment, and what would happen to me."

"(A parent said) What he/she cares about is if he/she is going to get surgery and if he/she needs continuous chemotherapy treatment... and he/she is curious about all these treatment procedures, and what will happen after a leg amputation."

"(A parent said) They ask if their children can survive through treatment, and when they hear cancer, they think it is almost as though their child is dying, like it's all over. So, what we do is we make them have hope for a cure, and hope can bring them out to take treatment actively."

"I think it's better to tell the truth honestly. I think being honest and giving positive additional explanations can work."

"Yes, what is the percentage or probability of survival; that's what I'm mostly curious about."

3) Medical terms

"Because you can be less anxious. It would be helpful if the medical staff let them know what they are doing. I didn't think of anything like this, but the medical terminology here is a great idea. Well, I don't know what is going to be like because it's all my personal opinion."

2. Information needs: Useful information during treatment (personal experience)

"The doctor basically tells me everything about it because I have to take care of myself and be aware of what to give and to eat. The doctor writes those figures down on paper and explains them all; what ANC is, why blood transfusions are needed when platelet counts fall, and how normal the white blood cell count should be, and so on. I think those things are basically coached in the hospital."

3. Support system: Useful support during treatment (personal experience)

1) Hospital school

"I was diagnosed with a first-year admission letter. That means I didn't get into elementary school. I was linked to the hospital school and was checked in attendance. The hospital school is linked to my elementary school, so my school year keeps going up."

"(A parent said) Oh, I think she is talking about a foundation like "Make Wish." There's a foundation that makes your one wish come true, like, well, letting patients meet their favorite idol. My kid has never been on a plane before so he really wanted to fly. But, you know, for limited expenses, you can't go abroad. So, our family went to Jejudo on a date right after the treatment was over, and he/she loved it. Hey, OO, the hospital school helped you too, didn't it?"

"Like the hospital school, or in-patient place to play, these kinds of programs helped us so much that the hospital life was not boring. I got really bored. But with these, it's less boring, and mothers can take a break. It helps a lot, with all those programs."

4. Barriers to treatment: Emotional reaction at the time of diagnosis and during treatment

1) At the time of diagnosis

"I didn't think about it, personally. I wasn't really interested in what disease I had got."

"(A parent said) I don't know if it's because we're in a cancer center, but he/she had this surgery and got a urine line for a while. The urine line was taken out, before long they said there's no more prescription so go home right away. I was really stuck. I said, crying, how could they tell us to leave already. My kid couldn't move her legs, and it hasn't been long since he/she took out the urine line."

"It wasn't pale, it became yellow. Those words really stabbed right into my heart. I thought, 'I've brought him/her to this point.' I cried hard hitting my chest, outside the Emergency Room, thinking I could have just run to a local clinic and ask for a CBC transfusion. I couldn't imagine a life after he/she died."

"I found out that it was cancer after reading the instruction papers, so I panicked."

2) During treatment

"I'm already anxious. When you're in the hospital, anything small like how to manage things, or like if you are in an emergency situation, you've got a place to contact I can ask, but when you're finished with the treatment….Well, my doctor [told] me it's fine to go back to school, as soon as possible, but I keep doubting if it's okay."

"(A parent said) Actually we've just passed six months since the treatment started. So you can say the period we've been through is not that long but not that short. It's somewhere in the middle, and we're about to end the treatment. I mean, there's still a lot

I don’t know about, but they say it’s going to be over shortly. I kept thinking about how and what are we going to prepare afterwards? The future just seems so vague. Of course, it happens during the course of the treatment. Most mothers who have been through a year and almost two years, they just come and go when they’re told by the hospital, and they just live their daily lives. They say that the fear is not that big, but rather that it’s comfortable when they stay in the hospital. So when I hear that, I feel rather frustrated. I have to go home now, and I have to take care of my kid at home myself, but I don’t know what to do.”

“This is the big problem. The reason I’m so supportive of this education program is that there’s a limitation of internet and you narrow down the range a little bit. Also, mothers can access to it too. I hope the contents go on in the future. It ends within eight weeks now, but I think there will be more and more content from now on.”

“I’d like you to include that certainly so that patients don’t get too nervous. I really thought bone marrow transplantations were really operating on your belly. I thought you were scratching it out of the bone.”

“We have a lot of fear not the testing itself, but before the testing, since the day before.”

5. Facilitators to treatment: Social support from friends, other patients, family members

1) Friends

“(A parent said) She didn’t tell us that much, because of this and that, but she went around openly telling her friends, ‘I’ve got cancer.’ She’s got this personality so he/she could do that. Then the friends thought it was like a disease you can easily get over like a cold. When teachers asked, ‘Does anybody know why OO is absent for a long time?’ and they said ‘He/she has cancer.’ By the time he/she was admitted to the hospital, a friend

who had already had the disease explained to us what we had to be careful about, drink pre-boiled water and other things in detail through KakaoTalk.”

“(friends’ supports) That’s why I felt very supportive, because I was really stuck as well.”

2) Other patients

“Just with the friends next to me were very helpful.”

“There was an older girl than me.”

3) Successful cases of previous patients

“I heard that it was nice to see a young man with the same diagnosis who was a survivor. Yes, it was a connection to a young man who was able to make his social life normal, who used to suffer from a childhood cancer and a transplant.”

II. User testing sessions: categories, themes, and feedback

1. Program content: Positive feedback on program content

1) Easy to understand, good readability

“It’s easy to understand if you read it with this (picture).”

2) Helpful and useful educational content

“First of all, there’s one… you can think of it as helpful to someone else, not to me. Although it’s not our case, if you’re suspicious that you might have brain tumors, you can visit a hospital. I think it does help.”

3) Knowledge/information first learned from this program

“(Participant reading the description) I’ve never heard of a prophylactic antibiotic injection.”

2. Program content: Negative feedback on program content

1) Content was not easy to understand, low readability (use of difficult medical terminology)

“Well, I get it from an adult’s perspective, but there are a lot of terms that kids might not comprehend.”

3. Program appearance: Positive feedback

1) Liked layout (e.g., font style, font size, sentence length, location of pictures and paragraphs)

“(Researcher said) If it’s hard to read because there’s too much content or there’s too much writing in one slide, you can tell me such a thing like that.”

“No, it’s fine. It’s all right.”

4. Program appearance: Negative feedback

1) Issues with layout (e.g., pictures sizes and location)

“Don’t you think it will be too small to see it on your phone? I think you should just turn the picture over to another page or something.”

5. Navigation: Positive feedback

1) Familiar with Facebook navigation

“Then if I don’t know the details right now, I think it would be helpful for patients to learn this by Facebook myself.”
