# Supplementary material for: Development and Usability Evaluation of a Facebook-Based Intervention Program for Childhood Cancer Patients: Mixed Methods Study
Source: J Med Internet Res. 2020 Jul 28;22(7):e18779. doi: 10.2196/18779 (PMC7420636; doi:10.2196/18779)

Appendix 2. Healthy Teens for *Soaam* screenshots

Screenshot 1: Homepage and Module 8 about Family issues – It included links to short video clips about sibling issue that is mentioned in the module contents.

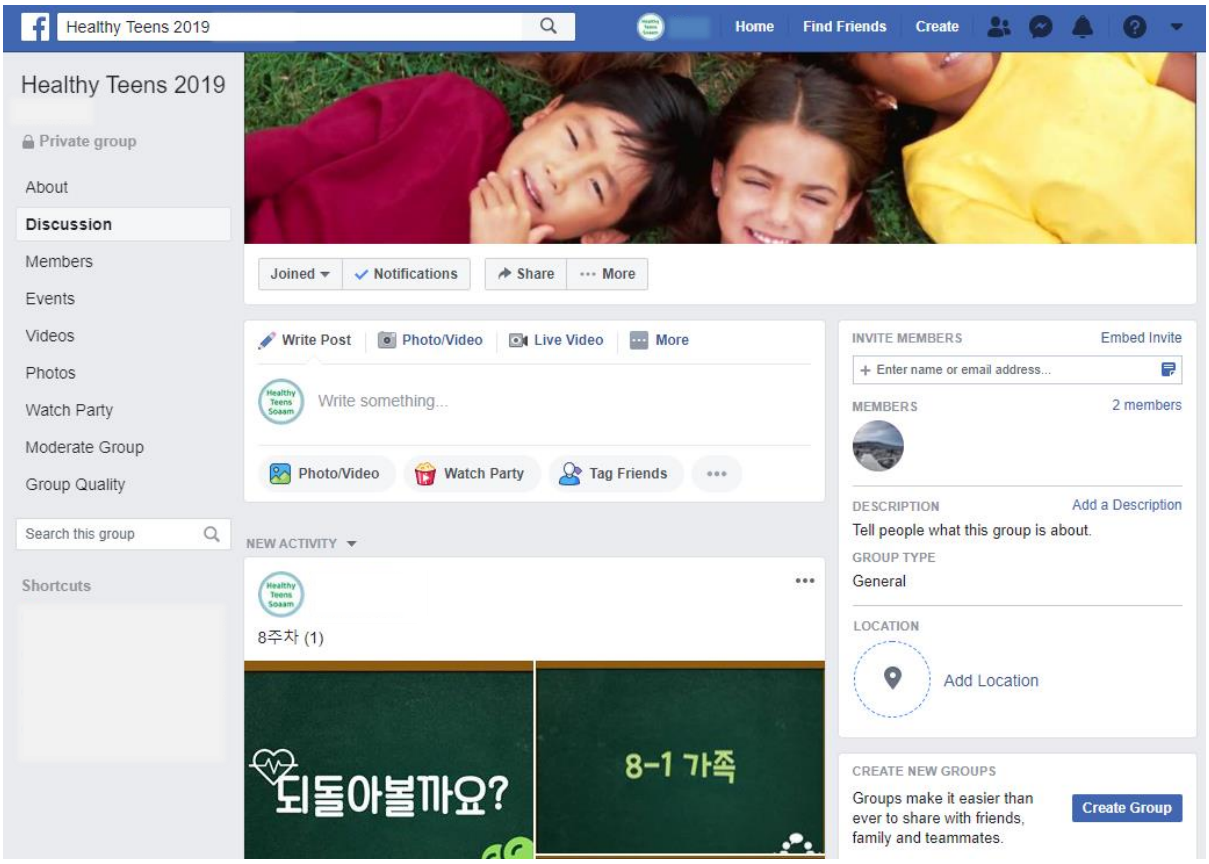

Screenshot 2-3: Module 3 about Diagnostic tests – It delivered information of imaging tests and included pictures and links to short video clips of taking MRI exam.

Screenshot 2

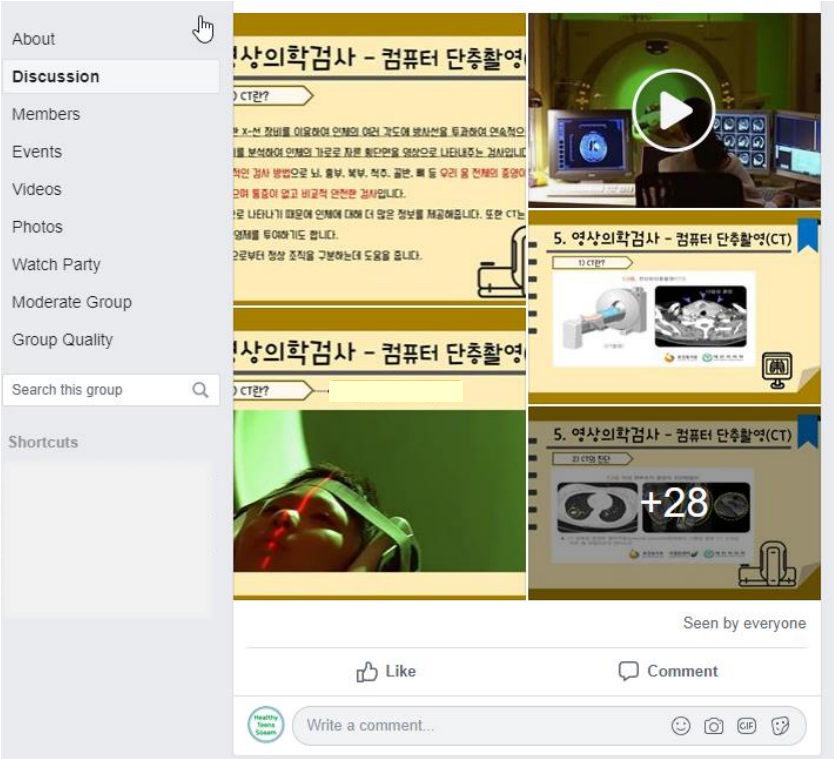

Screenshot 3

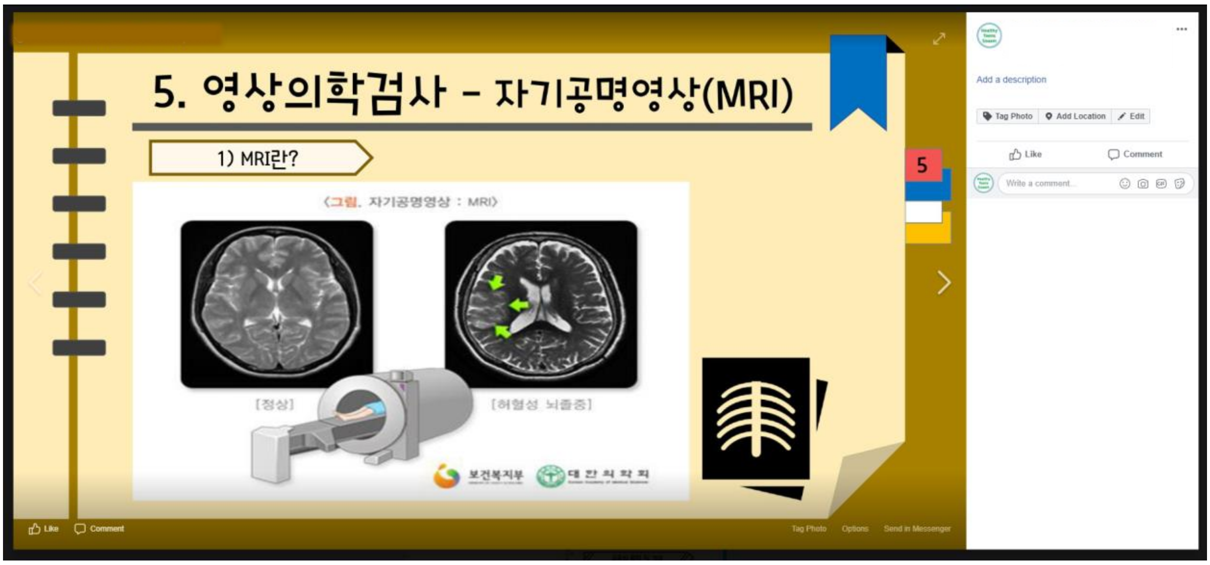

Screenshot 4: Module 2 about Types of pediatric cancers and their characteristics – It delivered information of various types of cancer with pictures.

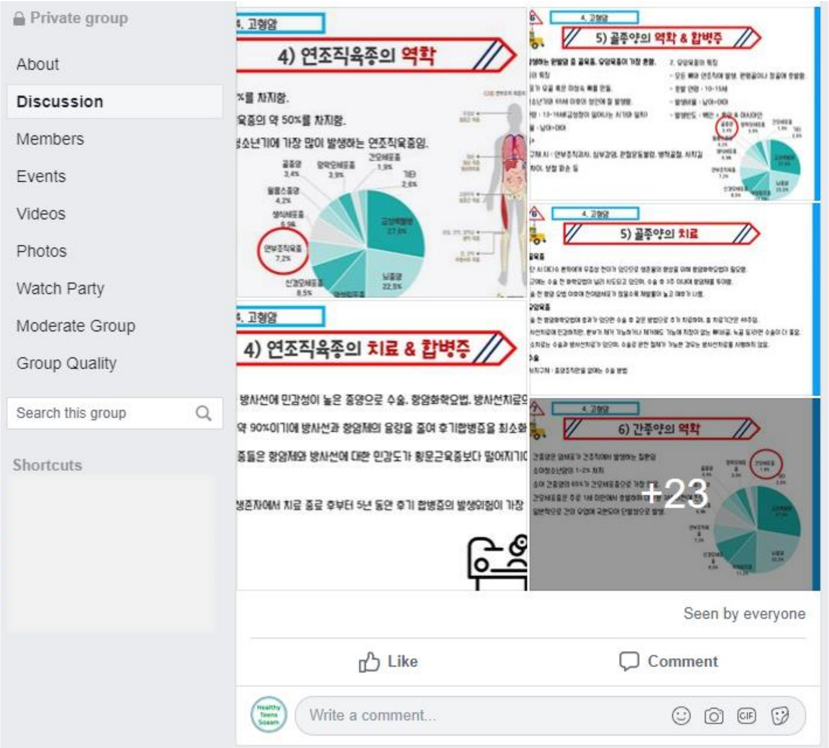

Supplement: Multimedia Appendix 2 [file jmir_v22i7e18779_app2.pdf]
